# The primary cilium functions as a mechanical and calcium signaling nexus

**DOI:** 10.1186/s13630-015-0016-y

**Published:** 2015-05-29

**Authors:** Kristen L Lee, Marie D Guevarra, An M Nguyen, Mardonn C Chua, Yingxiao Wang, Christopher R Jacobs

**Affiliations:** Department of Biomedical Engineering, Columbia University, 351 Engineering Terrace, MC 8904, 1210 Amsterdam Ave, New York, NY 10027 USA; Jacobs Technion-Cornell Innovation Institute, Cornell Tech, New York, NY 10011 USA; Department of Biotechnology, University of British Columbia, Vancouver, BC V6T 1Z4 Canada; Bioengineering Department, UC San Diego, La Jolla, CA 92093 USA

**Keywords:** Mechanotransduction, Primary cilium, Calcium signaling, Osteocyte, Kidney epithelia, Biosensor

## Abstract

**Background:**

The primary cilium is an antenna-like, nonmotile structure that extends from the surface of most mammalian cell types and is critical for chemosensing and mechanosensing in a variety of tissues including cartilage, bone, and kidney. Flow-induced intracellular calcium ion (Ca^2+^) increases in kidney epithelia depend on primary cilia and primary cilium-localized Ca^2+^-permeable channels polycystin-2 (PC2) and transient receptor potential vanilloid 4 (TRPV4). While primary cilia have been implicated in osteocyte mechanotransduction, the molecular mechanism that mediates this process is not fully understood. We directed a fluorescence resonance energy transfer (FRET)-based Ca^2+^ biosensor to the cilium by fusing the biosensor sequence to the sequence of the primary cilium-specific protein *Arl13b*. Using this tool, we investigated the role of several Ca^2+^-permeable channels that may mediate flow-induced Ca^2+^ entry: PC2, TRPV4, and PIEZO1.

**Results:**

Here, we report the first measurements of Ca^2+^ signaling within osteocyte primary cilia using a FRET-based biosensor fused to ARL13B. We show that fluid flow induces Ca^2+^ increases in osteocyte primary cilia which depend on both intracellular Ca^2+^ release and extracellular Ca^2+^ entry. Using siRNA-mediated knockdowns, we demonstrate that TRPV4, but not PC2 or PIEZO1, mediates flow-induced ciliary Ca^2+^ increases and loading-induced *Cox-2* mRNA increases, an osteogenic response.

**Conclusions:**

In this study, we show that the primary cilium forms a Ca^2+^ microdomain dependent on Ca^2+^ entry through TRPV4. These results demonstrate that the mechanism of mechanotransduction mediated by primary cilia varies in different tissue contexts. Additionally, we anticipate that this work is a starting point for more studies investigating the role of TRPV4 in mechanotransduction.

**Electronic supplementary material:**

The online version of this article (doi:10.1186/s13630-015-0016-y) contains supplementary material, which is available to authorized users.

## Background

Mechanotransduction is a process by which cells sense and convert mechanical signals into biochemical and transcriptional changes. The calcium ion (Ca^2+^) is a ubiquitous second messenger that regulates numerous signaling pathways. Despite how universal Ca^2+^ is, discrete intracellular signaling mechanisms occur because Ca^2+^ gradients are spatiotemporal and do not comprise one general pool that changes uniformly. For example, there are discrete microdomain Ca^2+^ signals including “Ca^2+^ sparks,” “Ca^2+^ sparklets,” and “scraps” that modulate constriction and relaxation in vascular smooth muscle cells [1,2]. Mechanical loading generates rapid and temporal intracellular Ca^2+^ increases in many cell types including osteocytes, osteoblasts, neurons, and kidney cells. Ca^2+^ mobilization is required for flow-induced prostaglandin E2 (PGE_2_) release and flow-induced osteopontin gene regulation in osteocytes, demonstrating that Ca^2+^ is upstream of mechanotransduction activities and paracrine signaling [3,4].

The primary cilium is an antenna-like, nonmotile structure that extends from the surface of most mammalian cell types into the extracellular space [5,6]. While initially considered a vestigial structure, in the past decade, several labs have demonstrated that primary cilia are critical for chemosensing and mechanosensing in a variety of tissues including cartilage, bone, and kidney [7-13]. The osteocyte primary cilium deflects with mechanical stimulation and mediates mechanotransduction at the transcriptional level; however, previous experiments have not resolved Ca^2+^ within the primary cilium from the cytosol [14].

Recent advances in monitoring ciliary Ca^2+^ mobilization have improved our understanding of the primary cilium-mediated mechanism of mechanotransduction in kidney epithelia. In the past, traditional diffusive BAPTA-based fluorescent indicator dyes were used to measure intracellular Ca^2+^ levels but did not target specific subcellular domains. In some pivotal studies, Praetorious and Spring and Nauli et al. demonstrated that primary cilia are required for mechanically induced Ca^2+^ increases in kidney epithelial cells [12,13,15]. The dependence of flow-induced Ca^2+^ increases on kidney epithelia primary cilia and the presence of mechanosensitive Ca^2+^-permeable channels on the ciliary membrane suggest that mechanical loading opens stretch-activated ion channels on the primary cilium that mediate Ca^2+^ entry. In the last couple of years, Delling et al., Su et al., and Jin et al. directed genetically encoded single fluorescence Ca^2+^ biosensors to the primary cilium using a variety of ciliary targeting sequences in human retina pigmented epithelia and kidney epithelial cells [16-18]. Su et al. and Jin et al. exposed kidney epithelial cells to fluid flow, which bent primary cilia and increased ciliary and cytosolic Ca^2+^ levels [17,18]. The Ca^2+^-permeable channel polycystin-2 (PC2) associates with the mechanosensitive protein polycystin-1 and localizes to the primary cilium. Jin et al. reported that flow-induced Ca^2+^ elevations occur first in the primary cilium and are followed by cytosolic Ca^2+^ mobilization. Both ciliary and cytosolic Ca^2+^ increases were dependent on PC2 [18]. Furthermore, blocking ryanodine receptors inhibited cytosolic Ca^2+^ increases without affecting the flow-induced ciliary Ca^2+^ response [18,19]. Collectively, these recent flow studies on kidney epithelia primary cilia demonstrate that fluid flow activates PC2 through which extracellular Ca^2+^ enters and triggers ryanodine receptors in Ca^2+^-induced Ca^2+^ release.

Current knowledge of the osteocyte primary cilium-mediated mechanism of mechanotransduction is relatively poor compared with recent progress in kidney epithelia primary cilium mechanotransduction research. Our group previously used the fluorescent dye Fura 2-AM to demonstrate that flow-induced Ca^2+^ increases in MLO-Y4 osteocyte-like cells are independent of primary cilia and stretch-activated channels, which is different from kidney cells [14]. While these results suggest that the osteocyte primary cilium-regulated mechanism of mechanotransduction is not linked to intracellular Ca^2+^ levels, it is unknown if the local primary cilium Ca^2+^ environment is distinct from the cytosol. We hypothesized that the osteocyte primary cilium mediates mechanotransduction by forming a distinct Ca^2+^ microdomain. Therefore, the objective of this study was to monitor flow-induced ciliary Ca^2+^ levels and elucidate the intricate role of the osteocyte primary cilium as a biochemical and mechanical signaling nexus.

In this study, we directed a fluorescence resonance energy transfer (FRET)-based Ca^2+^ biosensor to the primary cilium by fusing a biosensor sequence to the sequence of the primary cilium-specific protein *Arl13b*. The modified YC3.6 Ca^2+^-sensitive FRET-based biosensor with the ECFP-YPet donor-acceptor pair contains a calmodulin (CaM) region with four Ca^2+^-binding domains [20]. Binding of Ca^2+^ results in a conformational change that increases FRET signal, which is characterized by decreased ECFP and increased YPet fluorescence [21]. YPet as an acceptor produces the largest FRET dynamic range in live mammalian cells compared to Citrine, Venus, or cpVenus [20]. Using a targeted version of a FRET-based Ca^2+^ biosensor containing ECFP and YPet and the diffusive Ca^2+^ indicator dye Fura Red, we detected ciliary and cytosolic Ca^2+^ increases within individual MLO-Y4 cells exposed to fluid flow stimulation. Additionally, we examined the role of several Ca^2+^-permeable channels on the primary cilium: PC2, transient receptor potential vanilloid 4 (TRPV4), and PIEZO1. Coste et al. recently characterized mechanically activated current in neuroblastoma cells and proposed that the multipass transmembrane ion channels PIEZO1 and PIEZO2 mediate mechanically activated cation activity, leading us to explore the role of PIEZO channels in 223osteocyte mechanotransduction [22]. Our data demonstrate that TRPV4, but not PC2 or PIEZO1, mediates flow-induced ciliary Ca^2+^ increases and a loading-induced osteogenic response at the transcriptional level. Collectively, our study demonstrates that the osteocyte primary cilium microdomain is distinct from the cytosol and that sources of loading-induced ciliary Ca^2+^ mobilization are different in kidney epithelia and osteocytes. These are the first measurements of Ca^2+^ signaling within the osteocyte primary cilium, and we anticipate this work is a starting point for more studies investigating the role of TRPV4 in osteocyte mechanotransduction [23].

## Methods

### Plasmid construction

Drs. Yingxiao Peter Wang and Mingxing Ouyang previously developed a calcium-sensitive FRET-based biosensor composed of an ECFP donor, calmodulin region, M13 calmodulin-binding region, and YPet acceptor (CaB). Drs. Kenji Kontani and Kristen Verhey generously shared with us the *Arl13b* gene. We fused *Arl13b* to the N terminus of CaB using a 15-amino-acid-long flexible linker to form Arl13b-L-CaB (ALC)[24]. Deletions of Trp^3^ and Phe^17^ in the M13 region were performed to block changes in FRET during Ca^2+^ increases, serving as negative controls of CaB and ALC (mutCaB and mutALC) [25].

### Cell culture and transfection

MLO-Y4 osteocyte-like cells (a gift from Dr. Lynda Bonewald of the University of Missouri-Kansas City) were cultured in MEM alpha (Life Technologies) with 5% FBS, 5% CS, and 1% PS at 37°C in 5% CO_2_. Using the BTX 360 with a 300-V, 100-Ω, 1,000-μF pulse, 1.25 million cells were transfected with 10 μg plasmid by electroporation. Cells were co-transfected with the ALC plasmid and 0.5 nmol siRNA. siRNA sequences included Pkd2 (5′-CCUCUUGGCAGUUUCAGCCUGUAAA-3′), Trpv4 (5′-GAUGGACUGCUCUCCUUCUU GUUGA-3′), and Piezo1 (5′-CACCGGCAUCUACGUCAAAUA-3′) [22]. Transfected MLO-Y4 cells were seeded onto collagen I-coated glass slides (#1.5 glass, Warner Instruments) at a density of 4,000 cells/cm^2^ and cultured for 3 days in reduced serum containing 2.5% FBS and 2.5% CS. IMCD cells (purchased from ATCC®, CRL-2123™) were cultured in DMEM (Life Technologies) with 10% FBS and 1% PS. Transfected IMCD cells were seeded onto fibronectin-coated glass slides at a density of 8,000 cells/cm^2^ and cultured for 3 days in 1% FBS. Prior to imaging, cells were incubated in 20 μM Fura Red-AM (Life Technologies, F-3021) with 0.1% Pluronic® F-127 (20% solution in DMSO) (Life Technologies) for 1 h at room temperature to label cytosolic Ca^2+^.

### Imaging flow chamber

Slides were placed in the RC-30 Confocal Imaging Chamber (Warner Instruments) and attached to a syringe containing phenol red-free alpha MEM with 1% FBS and 1% CS for MLO-Y4 cells or phenol red-free DMEM with 1% FBS and 1% PS for IMCD cells. MLO-Y4 cells were stimulated with oscillatory fluid flow resulting in a 10-dyn/cm^2^ shear stress, and IMCD cells were stimulated with steady fluid flow resulting in a 1-dyn/cm^2^ shear stress, both within physiologic range for each cell type. Thapsigargin was added to the imaging media at a final concentration of 10 μM for appropriate samples for a 20-min rest period and maintained at the same concentration during flow. A separate syringe with 10 μM ionomycin was connected to the chamber to verify cell viability after the initial flow stimulus.

### Ca^2+^ imaging in cilia and cytosol

One fluorescing cell per slide was selected at random for imaging. The selected cilium was focused on, with the cell body captured within the field of view on an Olympus IX71 inverted epifluorescence microscope with a 1.30 N.A. ×40 oil immersion objective. Donor excitation was achieved on a xenon lamp using a 430/24-nm filter while images were collected simultaneously using a Quad-View system (QV2, Photometrics) with emission filters of 470/28, 530/30, and 641/75 nm. Cells were left to rest for 20 min prior to imaging. For calibration studies, we added ionomycin at a final concentration of 5 μM and CaCl_2_ at a final concentration of 0.1, 0.25, and 0.5 mM. Baseline signal was recorded for 30 s followed by 5 min of flow. Images were taken at 4 Hz with a 150-ms exposure.

### Image analysis

Images of each sample were trimmed to regions of interest that tightly enclosed the primary cilium or cell during imaging. Using MATLAB, images were corrected for background and bleedthrough and a pixel-by-pixel basis, resulting in signal only in the cilium and cytosol. Specifically, using MetaMorph, the same background region for ECFP and YPet images was selected from the untrimmed image far from the sample within the field of view, and bleedthrough coefficients were calculated prior to performing experiments [26]. To aid in peak detection, the average intensity data of a region of interest over time was smoothened using a 1D Savitsky-Golay filter with a window width of 31, and a fourth-degree smoothing polynomial. Oscillation amplitude was identified by finding local maxima with MATLAB. A baseline value for each sample was determined by averaging the baseline signal collected 30 s prior to flow. The maximum baseline oscillation amplitude was defined as maximum oscillation amplitude that occurred during the recording of the baseline signal, 30 s prior to flow. The Ca^2+^ peak height was defined as the peak amplitude that occurred during flow exposure. Multiple Ca^2+^ peaks may occur during the 5-min period of flow. A sample was considered responsive if a Ca^2+^ peak height was equal to or greater than 1.5 times the maximum baseline oscillation amplitude [27,28]. Not all samples were analyzed due to movement of the sample out of plane.

### Antibodies

We used the following primary antibodies: rabbit anti-polycystin-2 (Santa Cruz, sc-25749), rabbit anti-sera to TRPV4 (generously provided by Heller group), rabbit anti-PIEZO1 (Novus NBP1-78537 for immunostaining and NBP2-10504 for Western blot), mouse anti-acetylated alpha tubulin (Abcam, ab24610), and mouse anti-actin (Abcam, ab11003). We used the following secondary antibodies: Alexa Fluor 488 goat anti-rabbit IgG (Life Technologies, A11008), Alexa Fluor 568 goat anti-mouse IgG (Life Technologies, A11031), goat anti-rabbit IgG-HRP (Santa Cruz, sc-2004), and HRP goat anti-mouse Ig (BD Biosciences, 554002).

### Immunocytochemistry and confocal microscopy

MLO-Y4 and IMCD cells were seeded on 35-mm glass-bottom dishes at approximately 1,000 and 2,000 cells/cm^2^, respectively. Upon reaching 80%–90% confluence after 2 days of culture, cells were fixed with 10% formalin, permeabilized with 0.1% Triton X-100, and blocked with 10% goat serum and 1% BSA in PBS. Cells were labeled with primary antibodies for PC2, TRPV4, PIEZO1, and acetylated alpha tubulin followed by incubation in appropriate Alexa Fluor-labeled secondary antibodies. Nucleic stain was achieved using DAPI (0.5 mg/mL; 1:100). Confocal z-stack images were obtained on a Leica SP5 using a 1.46 N.A. ×100 oil immersion objective. Maximum X-Z projections were constructed with Leica software.

### Western blot

Cells transfected with *Pkd2*-, *Trpv4*-, *Piezo1*-, and MedGC-scrambled siRNA (Life Technologies) were lysed in RIPA buffer (Santa Cruz, sc-24948) supplemented with sodium orthovanadate, PMSF, and protease inhibitor cocktail 3 days post electroporation. Five micrograms of each protein sample was run through NuPAGE® Novex® 4%–12% Bis-Tris gels (Life Technologies). After electrophoresis, proteins were transferred to Invitrolon™ PVDF membranes (Life Technologies). The membranes were cut in half to separately label actin bands. Membranes for PC2, PIEZO1, and actin were blocked with 5% BSA (Sigma-Aldrich), and membranes for TRPV4 were blocked with 5% nonfat milk. HRP-conjugated antibodies were detected with chemiluminescence (Clarity Western ECL Substrate, Bio-Rad) on a Fujifilm LAS-4000 biomolecular imager.

### Flow chamber

Transfected MLO-Y4 cells were seeded onto collagen I-coated glass slides at approximately 5,000 cells/cm^2^ and cultured for 3 days. Slides were placed in larger, custom-made parallel-plate flow chambers (56 × 24 × 0.28 mm) so that sufficient amounts of RNA are isolated for gene expression analysis. Chambers with slides were incubated for 30 min and then exposed to 5 min of oscillatory fluid flow (OFF) at 1 Hz with a peak shear stress of 10 dyn/cm^2^. Slides were removed from chambers after 55 min, and RNA was isolated immediately.

### Quantitative real-time RT-PCR

RNA was extracted from cells using TriReagent (Sigma-Aldrich) and isolated, followed by cDNA synthesis using TaqMan reverse transcriptase (Applied Biosystems). cDNA samples were amplified with *Trpv4* (Mm00499025_m1), *Pkd2* (Mm00435829_m1), *Piezo1* (Mm01241570_g1), *Piezo2* (Mm01262433_m1), *Cox-2* (Mm00478374_m1), and *Gapdh* (4352339E) primers and probes (Applied Biosystems) by quantitative real-time RT-PCR using the ABI PRISM 7900 detection system (Applied Biosystems). Samples and standards were run in triplicate and were normalized to the endogenous *Gapdh* expression. Relative gene levels between samples were determined using the relative standard curve method (ABI Prism 7700 User Bulletin 2; Applied Biosystems).

### Statistical analysis

Results are shown as mean ± SEM. Unpaired *t*-tests (two-tailed) were used to analyze differences between treated and untreated groups. Comparisons of multiple groups were performed using one-way ANOVA followed by Dunnett’s multiple comparison post hoc test or Bonferroni’s multiple comparison post hoc test for *Cox-2* mRNA level comparisons. For all tests, *p* < 0.05 was considered significant.

## Results

### Arl13b-linker-Ca^2+^ biosensor detects ciliary Ca^2+^

**Figure 1:**
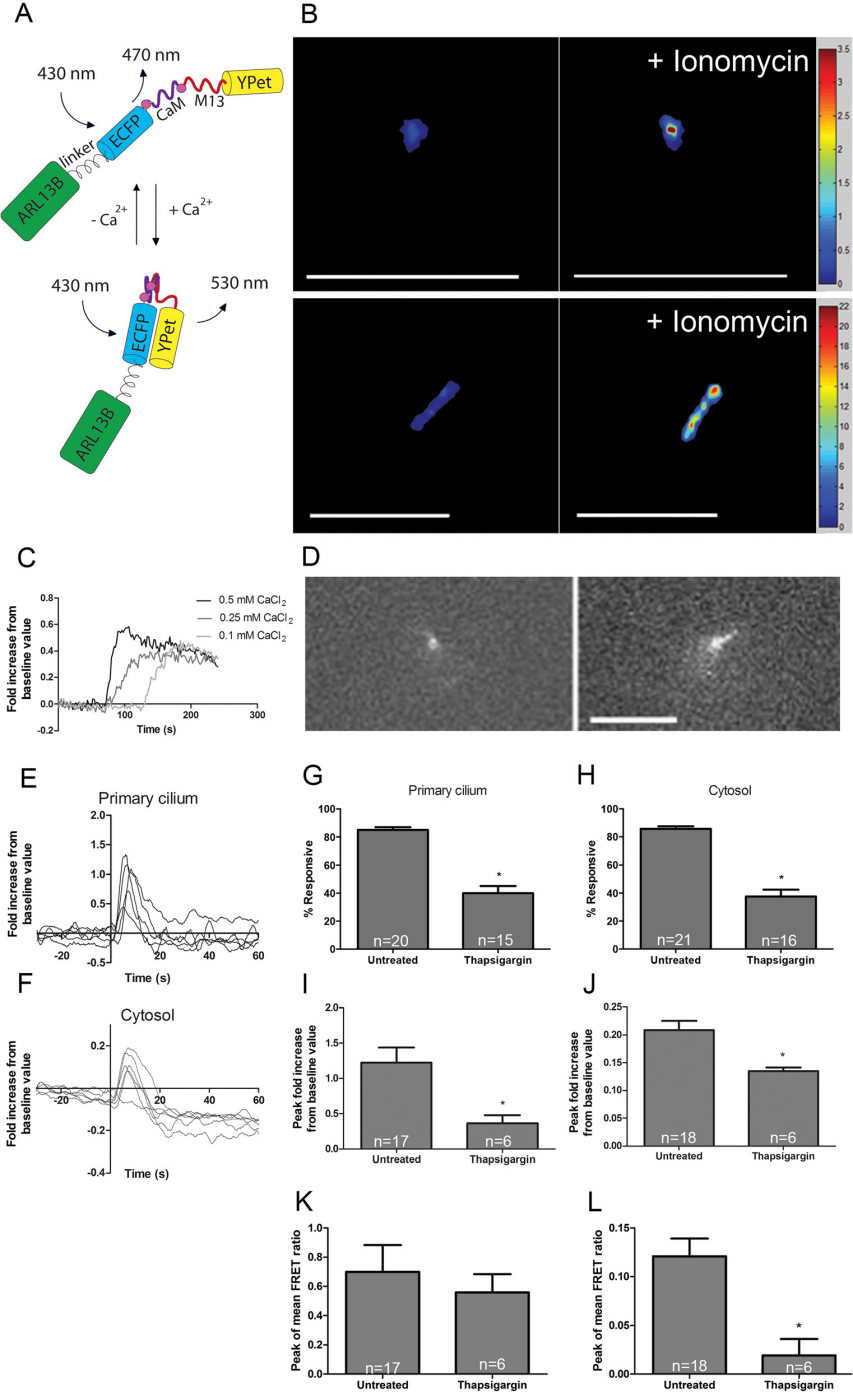
ALC localizes to the primary cilium and detects changes in ciliary Ca^2+^ levels. **(A)** Schematic of primary cilium-localized biosensor, ALC. **(B)** ALC FRET signal increases when ionomycin is added to media. FRET signal is reported on a pixel-by-pixel basis. Scale bars, 5 μm. **(C)** Traces of baseline-normalized FRET signal over time with different [CaCl_2_]. **(D)** YPet fluorescence images of a vertical primary cilium which bent during flow. Scale bars, 10 μm. **(E** **,** **F)** Individual ciliary and cytosolic Ca^2+^ measurements during oscillatory fluid flow starting at *t* = 0 s. **(G** **,** **H)** Percent of viable MLO-Y4 primary cilia and cytosolic domains exhibiting flow-induced Ca^2+^ increases (*n* = 15–21). **(I** **,** **J)** Flow-induced Ca^2+^ increases normalized to baseline value in the primary cilium and cytosol with untreated or thapsigargin-treated media (untreated (*n* = 17 or 18), thapsigargin (*n* = 6)). **(K** **,** **L)** Average peak response over baseline value for cilia and cytosolic domains with thapsigargin treatment for all viable cells (untreated (*n* = 17 or 18), thapsigargin (*n* = 6)). Error bars show ±SEM (*p < 0.05).

For this study, we developed a novel primary cilium-localized, fully ratiometric biosensor using the modified YC3.6 Ca^2+^-sensitive FRET-based biosensor (CaB) containing ECFP and YPet fused to ARL13B. First, we established that ARL13B localizes to primary cilia in MLO-Y4 osteocyte-like cells and verified ARL13B localization to IMCD primary cilia (Additional file 1: Figure S1A and B) [29]. Our biosensor design consisted of *Arl13b* at the N terminus followed by a 15-amino-acid-long flexible linker, ECFP, calmodulin, M13 calmodulin-binding region, and YPet at the C terminus (Figure 1A) [20,24]. Addition of Ca^2+^ leads to increased FRET signal, and transfecting Arl13b-linker-CaB (ALC) enabled us to detect Ca^2+^ levels within the primary cilium separate from the cytosol. The addition of 5 μM ionomycin, a Ca^2+^ ionophore, in media containing 1.8 mM Ca^2+^ led to detectable increases in FRET (represented by the emission ratio of YPet:ECFP fluorescence intensity) (Figure 1B, Additional file 2: Video S1). Furthermore, the FRET signal increased at a slower rate and with delay to smaller concentrations of calcium chloride (CaCl_2_) added with ionomycin in Ca^2+^-free media compared with higher concentrations of CaCl_2_ (Figure 1C). As expected with a *K*_d_ = 250 nM, the biosensor activity eventually reaches saturation for all CaCl_2_ concentrations. Thus, we determined that ALC is sensitive to different levels of ciliary Ca^2+^.

### Mechanical stimulation of MLO-Y4 cells leads to ciliary and cytosolic Ca^2+^ mobilization

We preferentially imaged cells with primary cilia having a vertical component prior to flow which typically bent in the direction of flow (Figure 1D). We loaded the Ca^2+^ indicator dye Fura Red into cells transfected with ALC to monitor cytosolic Ca^2+^ levels. Use of a Photometrics Quad-View beam splitter allowed us to collect images through several emission filters, and we monitored ECFP, YPet, and Fura Red signals simultaneously [30]. Application of 1 Hz OFF resulting in a 10-dyn/cm^2^ surface shear stress led to ciliary and cytosolic Ca^2+^ peaks within approximately 8.2 ± 0.8 and 8.2 ± 0.6 s, respectively (Figure 1E, *n* = 17 or 18). On an individual cell basis, 57% of Ca^2+^ peaks occurred in the primary cilium prior to the cytosol of the same cell. We also stimulated cells transfected with the inactive biosensor mutALC to verify that the observed peaks during 10 dyn/cm^2^ OFF were indeed due to increased Ca^2+^ levels (Additional file 3: Figure S2). Next, cells were treated with thapsigargin, which depletes intracellular Ca^2+^ stores (*n* = 6). As expected, we found that Ca^2+^ release from internal stores is a substantial source of flow-induced Ca^2+^ mobilization in the cytosol. Under thapsigargin treatment, there was a 45% decrease in responsive samples and a 25% magnitude reduction averaged from those fewer responsive samples in response to flow (Figure 1G, H). Additionally, under thapsigargin treatment, the peak mean signal from all viable samples in the group was significantly reduced in the cytosol but not in the primary cilium (Figure 1K, L). We continued to observe flow-induced ciliary Ca^2+^ peaks, although there were significant decreases in responsiveness and peak magnitude normalized to baseline value (Figure 1G–J). These results imply that Ca^2+^-permeable channels on the primary cilium also mediate flow-induced Ca^2+^ entry.

### PC2, TRPV4, and PIEZO1 are Ca^2+^-permeable channels on the primary cilium

**Figure 2:**
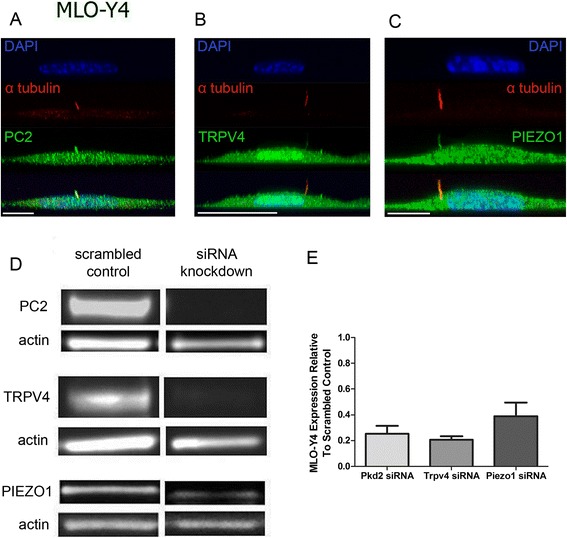
PC2, TRPV4, and PIEZO1 localize to MLO-Y4 primary cilia and plasma membrane. **(A** **–** **C)** Localization of PC2, TRPV4, and PIEZO1 on MLO-Y4 primary cilia and plasma membrane. Acetylated alpha-tubulin is in enriched primary cilia. Scale bars, 10 μm. **(D)** Chemiluminescence-detected Western blot of PC2, TRPV4, PIEZO1, and actin protein in MLO-Y4s treated with scrambled control siRNA and *Pkd2*, *Trpv4*, or *Piezo1* siRNA. **(E)** Quantified knockdown of *Pkd2* (*n* = 4), *Trpv4* (*n* = 10), and *Piezo1* (*n* = 4) mRNA expression in MLO-Y4 cells treated with siRNA relative to controls (*n* = 10). Error bars show ±SEM.

We examined three Ca^2+^-permeable channels localized to the osteocyte primary cilium: PC2, TRPV4, and PIEZO1. PC2 and TRPV4, among other channels, localize to primary cilia and mediate flow-induced Ca^2+^ increases in kidney epithelia [15,31]. Coste et al. reported that mechanically activated ion channels PIEZO1 and PIEZO2 are expressed in several murine tissues including kidney and have relatively lower expression in brain and heart tissues [22]. We observed *Piezo1* mRNA expression in MLO-Y4 and IMCD cells and *Piezo2 mRNA* expression only in IMCDs (Additional file 4: Figure S3). We immunostained MLO-Y4 cells for PC2, TPRV4, and PIEZO1 and found that all three channels are present in both the primary cilium and plasma membrane (Figure 2A–C). siRNA transfection reduced protein and mRNA levels compared with scrambled siRNA controls (all groups (*n* = 4–10) vs. control mRNA levels (*n* = 10), *p* < 0.005)( Figure 2D, E).

### TRPV4 mediates flow-induced ciliary Ca^2+^ increases in osteocytes

**Figure 3:**
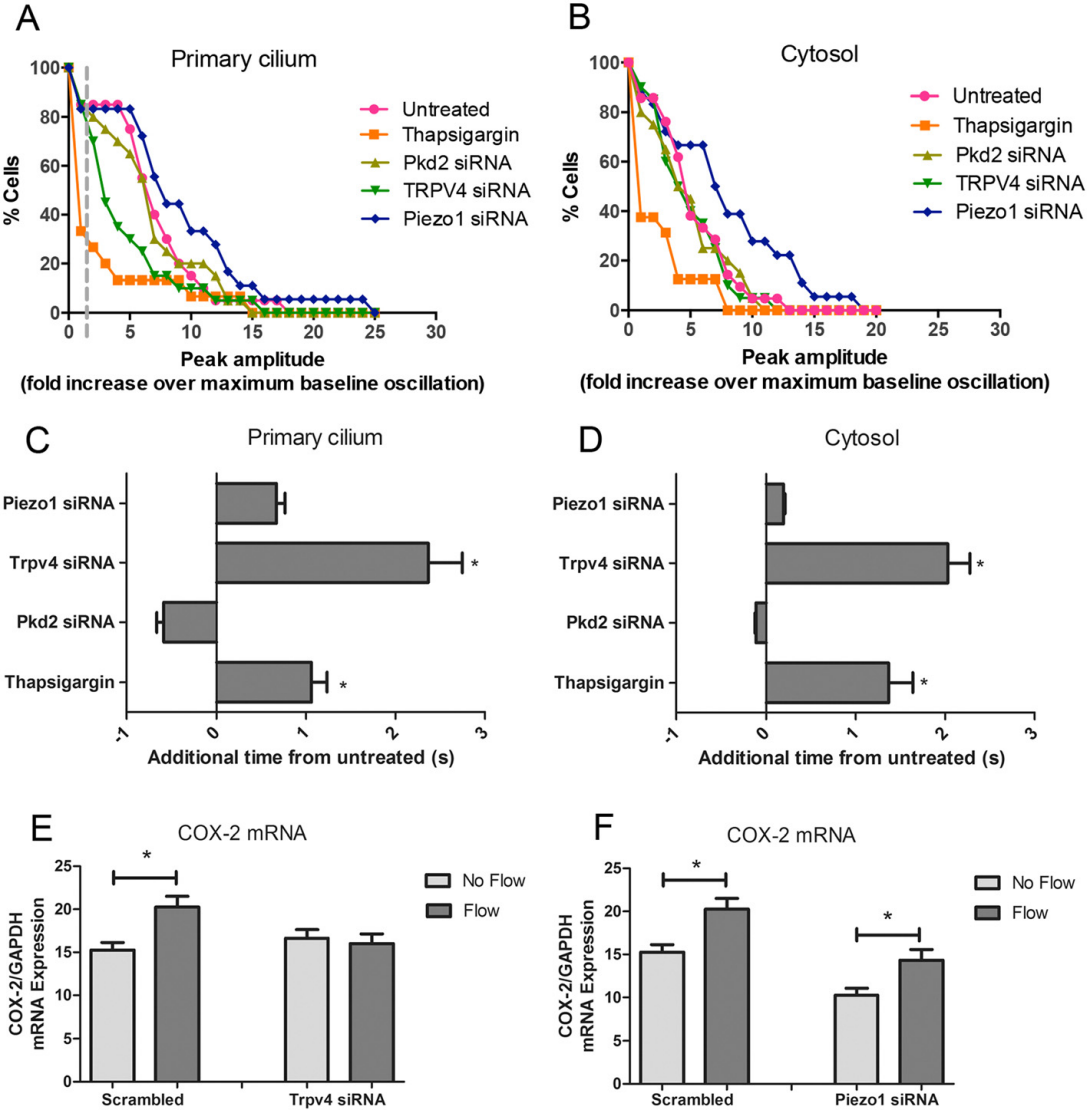
TRPV4 plays a role in osteocyte mechanotransduction. **(A,B)** Percent of viable cells exhibiting flow-induced Ca^2+^ response as a function of peak amplitude (a multiple of the sample’s maximum baseline oscillation amplitude) (*n* = 15–21). **(C** **,** **D)** Timing of ciliary and cytosolic Ca^2+^ peaks of treated cells relative to untreated controls (untreated (*n* = 15), thapsigargin (*n* = 5 or 6)), *Pkd2*, *Trpv4*, and *Piezo1* (*n* = 14–16). **(E** **,** **F)** Levels of normalized *Cox-2* mRNA expression in scrambled siRNA and *Trpv4* or *Piezo1* siRNA-treated cells with and without 5 min of oscillatory flow exposure (*n* = 9–11). Error bars show ±SEM (*p < 0.05).

PC2 mediates mechanically induced ciliary and cytosolic Ca^2+^ signaling in kidney epithelia, and here, our data suggest that osteocyte mechanotransduction is independent of PC2. Following co-transfection of ALC and either *Pkd2*, *Trpv4*, or *Piezo1* siRNA, MLO-Y4 cells were loaded with Fura Red and exposed to oscillatory flow stimulation (*n* = 14–16). Upon an initial general analysis of primary cilia from all viable cells (ionomycin-responsive), neither thapsigargin nor any of the siRNA-mediated knockdowns of *Pkd2*, *Trpv4*, or *Piezo1* exhibited a significant difference in flow-induced Ca^2+^ peak amplitude relative to untreated samples. Similarly, the only significant difference in peak amplitude relative to untreated samples occurred in the cytosol of cells treated with thapsigargin (Additional file 5: Figure S4). Using the spike threshold of 1.5× baseline, there was no significant difference in the percentage of cells exhibiting a Ca^2+^ response in the primary cilium and cytosol with *Pkd2*, *Trpv4*, and *Piezo1* knockdown. To illustrate the role of candidate mechanically activated channels in regulating Ca^2+^ entry in a different way, we plotted the percent of viable cells exhibiting flow-induced Ca^2+^ responses as a function of peak amplitude (a multiple of the sample’s maximum baseline oscillation amplitude) for all treatment groups. Thus, a flow-induced peak was considered a response if its amplitude was 1.5× greater than the maximum baseline oscillation amplitude, and we demonstrated that the percentage of cells responding decreased as a peak was considered a larger multiple of maximum baseline oscillation. Out of all the candidate Ca^2+^-permeable channels, only the loss of *Trpv4* clearly impaired the percent of responding cells (Figure 3A). *Trpv4* deficiency did not lower the percent of cells exhibiting cytosolic Ca^2+^ peaks (Figure 3B). Additionally, only thapsigargin treatment affected the percent of cells exhibiting flow-induced cytosolic Ca^2+^ increases (Figure 3B, *n* = 6). We analyzed the response of *Trpv4* siRNA-treated cells further and found that 75% of untreated cells had an intense response, a Ca^2+^ peak height at least five times the maximum baseline oscillation amplitude, compared with only 30% of *Trpv4* siRNA-treated cells that had intense ciliary Ca^2+^ peaks. *Pkd2* and *Piezo1* deficiencies did not inhibit the percent of ciliary or cytosolic Ca^2+^ peaks. Interestingly, *Piezo1* knockdown sensitized the cytosolic Ca^2+^ response to flow, perhaps because of a different Ca^2+^ permeable channel.

As mentioned earlier, there is a delay in peak FRET signal with lower Ca^2+^ concentrations relative to higher Ca^2+^ concentrations. Compared with untreated samples, ciliary and cystolic Ca^2+^ peaks were delayed in MLO-Y4 cells treated with thapsigargin or deficient in *Trpv4* and *Piezo1* (Figure 3C, D). These differences were not statistically significant in cells lacking *Piezo1*. On an individual cell basis, there was no significant change in the percent of peaks occurring in the primary cilium prior to the cytosol. 57 ± 5% of peaks first occurred in the primary cilium in untreated cells, compared with 75 ± 13% in thapsigargin-treated cells, compared with 54 ± 5% in *Pkd2* siRNA-treated cells, 43 ± 5% in *Trpv4* siRNA-treated cells, and 55 ± 6% in *Piezo1* siRNA-treated cells. Thus, in cells deficient in *Trpv4*, there was a decrease in the percent of Ca^2+^ peaks occurring first in the primary cilium in cells deficient in *Trpv4*, although this difference was not significant. Collectively, these results suggest that fluid flow mechanically activates TRPV4 channels on the primary cilium. Separately, fluid flow also activates intracellular Ca^2+^ release, which diffuses from the cytosol into the primary cilium microdomain.

### TRPV4 is required for osteocyte mechanotransduction

Next, we were interested in determining if TRPV4 also mediates osteocyte mechanotransduction at the transcriptional level in addition to regulating early ciliary Ca^2+^ mobilization. The *Cox-2* gene encodes an enzyme which catalyzes formation of PGE_2_, an osteogenic cytokine released by osteoblasts and osteocytes in response to mechanical stimulation, and *Cox-2* expression increases correspond to more PGE_2_ release [14]. After 5 min of OFF stimulation (identical to the imaging experiments) and a rest period of 55 min, we isolated and quantified *Cox-2* mRNA expression relative to the endogenous control *Gapdh* (*n* = 9–11). Scrambled siRNA-transfected cells demonstrated a flow-induced increase in *Cox-2* mRNA expression levels, and this flow-induced response was blocked in cells lacking *Trpv4* (1.3 ± 0.3-fold (control) vs. 1.0 ± 0.1-fold (*Trpv4*) increase) (Figure 3E). MLO-Y4 cells transfected with scrambled control siRNA and *Trpv4* siRNA expressed similar levels of *Cox-2* mRNA expression at baseline. Interestingly, *Piezo1* deficiency did not affect downstream flow-induced changes in *Cox-2* mRNA. MLO-Y4 cells transfected with *Piezo1* siRNA demonstrated a 1.4 ± 0.2-fold flow-induced increase in *Cox-2* mRNA expression which was not significantly different from controls, although they exhibited lower baseline *Cox-2* mRNA expression levels (Figure 3F). We did not examine the role of PC2 in flow-induced transcriptional changes due to the absence of ciliary and cytosolic Ca^2+^ peak amplitude or timing changes with *Pkd2* deficiency. Taken together, these data suggest that loss of *Trpv4* alters osteocyte mechanotransduction at the molecular and transcriptional levels.

### Primary cilium-regulated mechanisms of mechanotransduction are different in kidney epithelia and osteocytes

**Figure 4:**
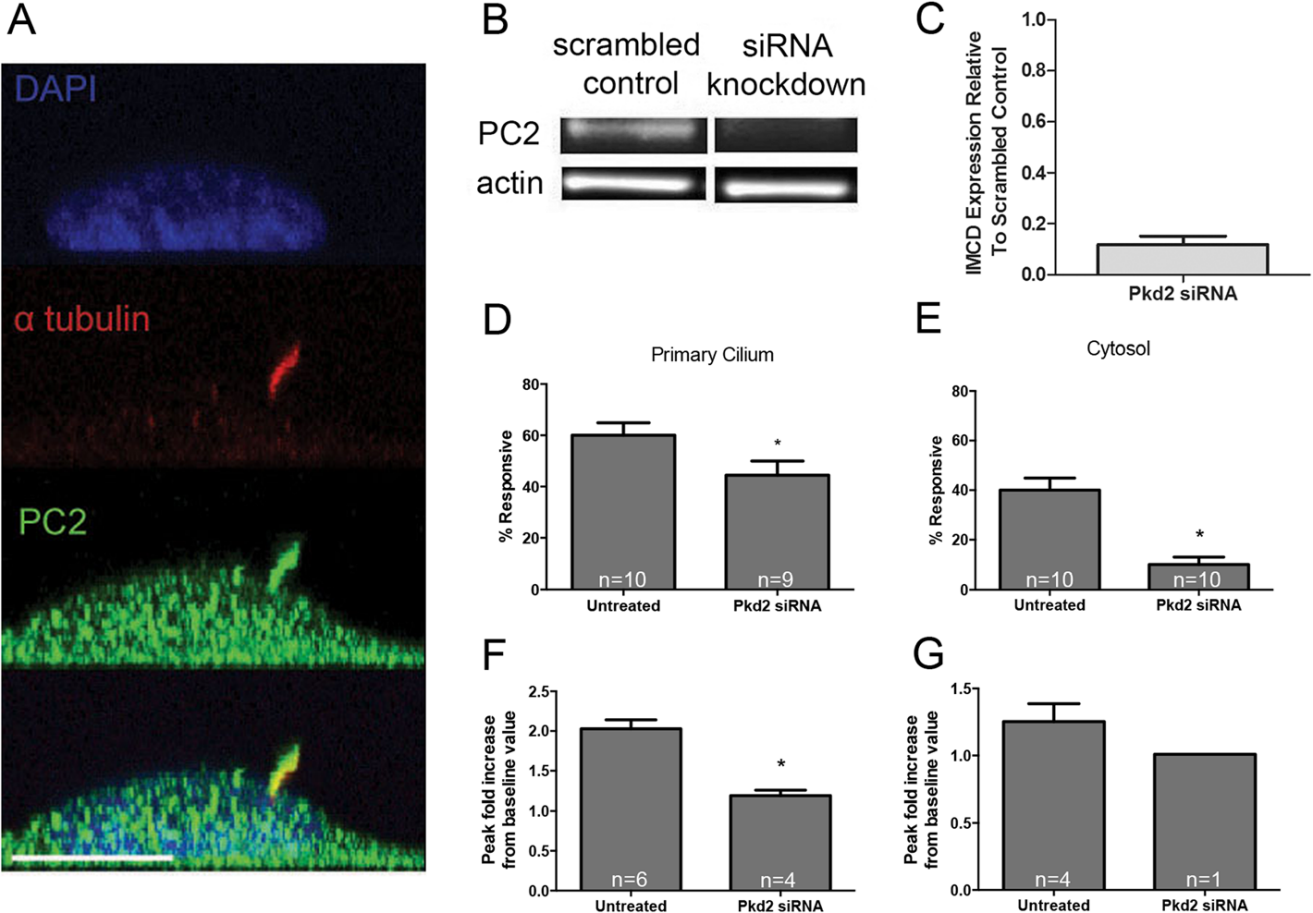
PC2 mediates kidney epithelial mechanotransduction. **(A)** PC2 localizes to IMCD primary cilia and plasma membrane. Scale bar, 10 μm. **(B)** Chemiluminescence-detected Western blot of PC2 and actin protein in IMCDs treated with scrambled control siRNA and *Pkd2* siRNA. **(C)** Quantified knockdown of *Pkd2* mRNA expression in IMCD cells treated with siRNA relative to controls (*n* = 4). **(D** **,** **E)** Percent of viable IMCD primary cilia and cytosolic domains exhibiting flow-induced Ca^2+^ increases (*n* = 9–10). Error bars show ±SEM (*p < 0.05). **(F** **,** **G)** Flow-induced Ca^2+^ increases normalized to baseline value in the primary cilium and cytosolic domains of responsive cells with *Pkd2* siRNA-mediated knockdown (primary cilium: untreated (*n* = 6), *Pkd2* (*n* = 4); cytosol: untreated (*n* = 4), *Pkd2* (*n* = 1)).

With the suggestion that the osteocyte primary cilium-mediated mechanism of mechanotransduction depends on TRPV4 and not PC2, we conducted similar flow studies with kidney epithelial cells to verify that this difference was due to cell type and not the experimental approach. We immunostained IMCD cells for PC2 and found similar localization relative to MLO-Y4 cells (Figure 4A). As expected and previously reported, *Pkd2* knockdown (11.85 ± 0.03% relative to IMCD control mRNA levels, *p* < 0.005 (Figure 4B, C, *n* = 4)) blocked steady flow-induced ciliary and cytosolic Ca^2+^ increases in IMCDs normalized to baseline value, and these differences were statistically significant (Figure 4D–G) [18].

## Discussion

In this study, we directed a FRET-based Ca^2+^ biosensor to the primary cilium and loaded cells with a nontargeted fluorescent Ca^2+^ indicator dye to resolve the local Ca^2+^ environment in the osteocyte primary cilium from the cytosol. We monitored ciliary and cytosolic Ca^2+^ levels using an epifluorescence microscopy system and observed flow-induced Ca^2+^ peaks in both domains. *Trpv4* deficiency reduced flow-induced ciliary Ca^2+^ peaks but did not impair flow-induced cytosolic Ca^2+^ mobilization, illustrating that the primary cilium microdomain is distinct from the cytosol. Thapsigargin treatment impaired flow-induced ciliary and cytosolic Ca^2+^ peaks, demonstrating that intracellular Ca^2+^ release and separate Ca^2+^ entry through TRPV4 are both components of ciliary Ca^2+^ mobilization. In contrast, knockdown of *Pkd2* and *Piezo1* did not affect ciliary or cytosolic Ca^2+^ peaks. Last, we linked the role of TRPV4 in regulating flow-induced ciliary Ca^2+^ mobilization with a downstream osteogenic response at the transcriptional level by determining that flow-induced changes in *Cox-2* expression depend on TRPV4. Collectively, our study demonstrates that the loading-induced ciliary Ca^2+^ mechanism is different between kidney epithelia and osteocytes.

After observing flow-induced Ca^2+^ peaks in both the osteocyte primary cilium and cytosol, we were motivated to identify the source of the ciliary Ca^2+^ peak. To deplete intracellular Ca^2+^ stores, we treated cells with thapsigargin and continued to observe ciliary Ca^2+^ peaks, suggesting that Ca^2+^-permeable channels on the primary cilium have a role in mediating flow-induced Ca^2+^ entry. However, thapsigargin treatment did lower flow-induced ciliary and cytosolic Ca^2+^ peak magnitudes and responsiveness compared with untreated cells, indicating that intracellular Ca^2+^ release is a component of flow-induced ciliary Ca^2+^ mobilization in osteocytes. Furthermore, a statistically significant delay in ciliary and cytosolic Ca^2+^ peaks occurred in thapsigargin-treated cells compared with controls. Thus, our data demonstrate that intracellular Ca^2+^ release contributes, in part, to the local primary cilia Ca^2+^ environment and suggests that the primary cilium serves as an important signal integrator.

It is important to note that a general ensemble analysis of our data did not reveal an effect of PC2, TRPV4, or PIEZO1 on flow-induced ciliary Ca^2+^ mobilization. In fact, the only significant reduction in peak Ca^2+^ mobilization was demonstrated in the cytosol with thapsigargin treatment. On the one hand, although the effect of thapsigargin on flow-induced Ca^2+^ mobilization was clear, using such a potent agent provides limited details. As expected, extracting the finer details of the role of Ca^2^ channels in targeted Ca^2^ channel blocking experiments required more selective analysis, which included categorizing cells into responders and nonresponders. While selecting a discriminating threshold can be confounding to the analysis, we found no significant difference in average peak ciliary Ca^2+^ mobilization with any treatment, suggesting that the results of this approach are not sensitive to the magnitude of the threshold (Additional file 5: Figure S4).

In this study, we present evidence that fluid flow activates TRPV4 on the primary cilium membrane and mediates Ca^2+^ influx. Using immunocytochemistry techniques, we determined that the stretch-activated Ca^2+^-permeable channel TRPV4 localizes to the primary cilium and plasma membrane. Interestingly, TRPV4 (and PC2 and PIEZO1) appears throughout the cell, which is consistent with TRPV4 and PC2 immunostaining in the literature [32-35]. While the distribution of the channels appear higher on the cell membrane relative to the primary cilium and may mediate a larger Ca^2+^ flux compared with the flux into the primary cilium, it is likely that the machinery and spatiotemporal molecular pathways unique to the primary cilium play a role in downstream mechanotransduction. Our flow experiments revealed that *Trpv4* knockdown lowered flow-induced ciliary Ca^2+^ peaks but did not impair cytosolic Ca^2+^ peaks. Unlike kidney epithelia, where Ca^2+^ reportedly enters the primary cilium through PC2 and induces CICR via ryanodine receptor activation [18], *Trpv4* deficiency in osteocytes did not affect cytosolic Ca^2+^ mobilization. This result suggests that the TRPV4-mediated ciliary Ca^2+^ microdomain does not regulate CICR in osteocytes. This is different from astrocytes, where TRPV4-mediated CICR regulates neurovascular coupling in an IP_3_R-regulated system [36]. It is also possible that other mechanically activated Ca^2+^-permeable channels compensate for the loss of TRPV4 function in the cytosol, which is consistent with data from this study showing that the loss of *Piezo1* may sensitize cells to flow. Knockdown of PC2, TRPV4, and PIEZO1 channels did not impair the flow-induced cytosolic Ca^2+^ response, which suggests that a different mechanism is in play that maintains normal cytosolic Ca^2+^ levels. Taken together, TRPV4’s location in an area of high membrane strain on the primary cilium and dependence of the flow-induced ciliary Ca^2+^ peak on TRPV4 suggest that the primary cilium acts as a Ca^2+^ and mechanical signaling nexus dependent on TRPV4.

Consistent with these results, previously, Malone et al. blocked flow-induced extracellular Ca^2+^ entry into MC3T3 osteoblasts using gadolinium chloride, which did not eliminate the flow-induced cytosolic Ca^2+^ flux [14]. In addition to demonstrating that flow-induced cytosolic Ca^2+^ flux is not dependent on extracellular Ca^2+^ entry through stretch-activated channels, Malone et al. also reported that the removal of primary cilia did not affect flow-induced cytosolic Ca^2+^ flux in osteocytes. The results in this study, here, are consistent with the previous paper and provide additional insight to the understanding of osteocyte mechanotransduction. Using advanced imaging techniques that provide enhanced resolution, our data demonstrate that flow-induced cytosolic Ca^2+^ flux is independent of extracellular Ca^2+^ entry through stretch-activated channels; furthermore, flow-induced ciliary Ca^2+^ flux is dependent on the Ca^2+^-permeable, stretch-activated TRPV4 channel. Thus, our data suggest that Ca^2+^ mobilization occurs differently in the cytosol versus the primary cilium during osteocyte mechanotransduction.

Our understanding of primary cilium bending mechanics and mechanical forces on the plasma membrane covering the primary cilium is essential to elucidating the molecular mechanism of mechanotransduction mediated by the primary cilium. Recently, Young et al. studied the tension force distribution along a primary cilium under flow and suggested that stretch-activated ion channels are likely to be activated and open near the base of the primary cilium where tension force is the highest [37]. While primary cilia bending is one potential physical event, it is possible that primary cilium deflection is not physiologic and that mechanical loading of the primary cilium occurs in other ways. For example, β-1 integrins are localized to MDCK primary cilia, and β-1 integrins have been implicated in mediating osteocyte mechanotransduction and loading-induced bone formation [38-40]. Thus, increased membrane tension is not limited to primary cilia bending and may involve primary cilia integrin-extracellular matrix interactions.

We anticipate that TRPV4 will be an attractive pharmacologic target for treating disuse-induced bone loss due to its role mediating osteocyte mechanotransduction and its sensitivity to existing biochemical agents (agonists: 4α-PDD, GSK1016790A, and RN1747 and antagonist: RN1734) [41-43]. Thus, treatment with TRPV4 agonists and therapies that elongate osteocyte primary cilia (lithium chloride, hydrogen sulfide, interleukin-1) to enhance mechanical strain levels may amplify osteogenic responses and prevent disuse-induced bone loss in patients restricted to bed rest [8,44,45]. Furthermore, O’Conor et al. have shown that TRPV4 plays a role as a physical transducer in chondrocytes, which may provide insight into functional cartilage tissue engineering approaches [46].

Other groups have suggested that TRPV4 is a candidate therapeutic target for bone loss disease. Mizoguchi et al. and Masuyama et al. have examined TRPV4 deficiency in unloading-induced bone formation, and they determined that *Trpv4* knockout reduces unloading-induced bone loss due to suppressed osteoclast numbers and impaired bone resorption [47,48]. Interestingly, Mizoguchi et al. do not exclude the possibility that *Trpv4* is expressed by osteocytes. Osteocytes are mechanosensing cells in bone that regulate osteoclasts’ resorption activities, and it will be important to understand the role of TRPV4 in the population of cells involved in mechanotransduction. The effect of an osteocyte-specific *Trpv4* deletion in loading-induced bone formation *in vivo* would provide evidence indicating whether TRPV4 is involved in the mechanotransduction process versus restricted to mediating osteoclast numbers and bone resorption. Additionally, it is important to recognize that primary cilia play important roles in development; however, in this study, we have not examined how TRPV4 plays a role in developmental ciliary pathways such as Hedgehog and Wnt signaling pathways.

While Jin et al. reported that flow-induced Ca^2+^ mobilization occurs in primary cilia before cytosolic Ca^2+^ increases in kidney epithelial cells, our flow studies do not demonstrate a clear difference in the timing between ciliary and cytosolic Ca^2+^ peaks in osteocytes [18]. While the amount of ciliary Ca^2+^ peaks occurring before cytosolic Ca^2+^ peaks is numerically higher than the number of ciliary Ca^2+^ peaks occurring after cytosolic Ca^2+^ peaks, it is unclear if ciliary Ca^2+^ triggers cytosolic Ca^2+^ increases. Unlike the side-dimension imaging method leveraged by Jin et al. to capture calcium signaling along the length of the primary cilium, our imaging method collected signal from only a part of the primary cilium that was in plane. Thus, we are unable to characterize a relationship between ciliary and cytosolic Ca^2+^ peak timing.

Several other groups have demonstrated that flow-induced ciliary Ca^2+^ mobilization is dependent on PC2 in kidney epithelia [15,18,31]. Our data suggest that flow-induced ciliary Ca^2+^ mobilization is dependent on TRPV4, and not PC2, in osteocytes. We conducted similar flow studies with kidney epithelial cells to verify that this difference was due to cell type and not the experimental approach. Our studies with IMCD cells also showed that flow-induced ciliary and cytosolic Ca^2+^ increases depend on PC2. The consistency in our results suggests that the mechanism of mechanotransduction mediated by primary cilia varies across different tissue contexts. Another difference between the MLO-Y4 and IMCD flow studies was the type of flow regime, consisting of OFF resulting in a 10-dyn/cm^2^ shear stress for MLO-Y4 cells and steady flow resulting in a 5-dyn/cm^2^ shear stress for IMCD cells. Previously, Malone et al. determined that flow-induced Ca^2+^ flux differences in MC3T3-E1 osteoblasts and MDCK kidney cells did not depend on these specific flow regimes, which suggests that primary cilium-mediated mechanosensation in osteoblasts and kidney cells is indeed different [14]. Thus, the application of different but physiologically relevant mechanical loads was appropriate for elucidating intricacies in the mechanotransduction mechanism in IMCD and MLO-Y4 cells.

Interestingly, in addition to being well-established in kidney epithelial mechanotransduction [15,18,31], PC2 has recently been implicated in osteocyte mechanotransduction [49]. The authors found siRNA-mediated knockdown of PC2 inhibited downstream flow-induced nitric oxide production and inducible nitric oxide synthase. While fluid flow-induced shear stress is known to activate the nitric oxide pathway, this activation occurs over a much longer time scale of hours compared to Ca^2+^ flux of seconds and minutes. Here, we studied the early signaling response to flow occurring within seconds after exposure to flow. Thus, our finding that PC2 was independent of mechanically induced ciliary and cytosolic Ca^2+^ signaling in osteocytes does not contradict the findings by Xu et al. It is possible that PC2 is involved in the later downstream signaling response to flow. While we found that PC2 may not be involved in the early Ca^2+^ response to flow, it is nonetheless an important channel in bone. *Pkd2* mutations have been implicated in skeletal development [50,51], and mutations in *Pkd1*, encoding the other subunit of the polycystin complex, have impaired both skeletal development and adaptation [52].

## Conclusions

In conclusion, this study highlights the specialization of primary cilium mechanisms across different tissue contexts. Here, we demonstrate that mechanically stimulated ciliary Ca^2+^ mobilization is different between kidney epithelia and osteocytes. Strikingly, the osteocyte primary cilium forms a distinct microdomain from the cytosol during mechanical loading. The primary cilium microdomain may help maintain spatiotemporal organization within the cell which allows numerous molecular mechanisms to occur with just a limited number of signaling molecules. We expect that the Ca^2+^-permeable channel TRPV4 will be an attractive therapeutic target for bone loss disease because of its location in an area of high membrane strain on the primary cilium, demonstrated role as a physical transducer in osteocytes in this study and chondrocytes in a recent study by O’Conor et al., and sensitivity to existing biochemical agents [46]. Furthermore, we anticipate that other flow studies on second messenger-mediated pathways in the primary cilium microdomain and loading-induced bone formation studies using mice with an osteocyte-targeted deletion of *Trpv4* will elucidate how TRPV4-mediated Ca^2+^ entry in the primary cilium microdomain regulates osteogenic responses to mechanical loads.

## **Additional files**

Additional file 1: Figure S1. ARL13B localizes to primary cilia in MLO-Y4 and IMCD cells. (A-B) IMCD and MLO-Y4 cells fixed and stained for acetylated alpha tubulin and ARL13B, demonstrating localization of ARL13B to the primary cilium. Additional file 2: Video S1. Ciliary Ca^2+^ increases detected by ALC. Images of an individual primary cilium in a time-lapse video. MLO-Y4 cells were transfected with ALC, cultured in 1.8 mM Ca^2+^-containing media, and treated with ionomycin which permeabilizes the plasma membrane to Ca^2+^. Images were captured at 10-s intervals. Display rate is three frames per second. Additional file 3: Figure S2. Mutant CaB and ALC do not exhibit flow-induced Ca^2+^ peaks. Deletions of Trp^3^ and Phe^17^ in the M13 region inhibit FRET changes during Ca^2+^ increases. (A-B) In initial testing and prior to targeting the mutated biosensor to the primary cilium, MutCaB failed to detect flow-induced Ca^2+^ increase with the addition of 10 μM CaCl_2_ (*n* = 3) and steady flow (5 dyn/cm^2^) (*n* = 2). (C) MutALC failed to detect flow-induced Ca^2+^ increases occurring with oscillatory fluid flow (10 dyn/cm^2^) (*n* = 4). Additional file 4: Figure S3. MLO-Y4 cells express Piezo1 and not Piezo2. (A) *Piezo1* and (B) *Piezo2* mRNA levels in MLO-Y4 cells, IMCD cells, and adult murine (C57BL/6) brain and heart relative to GAPDH mRNA expression (*n* = 4–5). *Piezo1 =* 1.6 ± 0.2 and *Piezo2* = 0.003 ± 0.001 in MLO-Y4 cells. Error bars show ±SEM. Additional file 5: Figure S4. FRET ratios of all viable MLO-Y4 cells exposed to fluid flow. (A) Mean FRET ratios over time, where solid lines indicate the mean background-corrected FRET ratios (no threshold applied) while the dashed lines indicate SEM. (B) Flow-induced Ca^2+^ peak amplitude averaged from all viable cells (no threshold applied). Error bars show ±SEM.

## Abbreviations

Cox-2: Cyclooxygenase 2
IMCD: Inner medullary collecting duct
PC-2: Polycystin-2
TRPV4: Transient receptor potential vanilloid 4
CaB: Calcium biosensor
ALC: Arl13b-linker-calcium biosensor
