# Supplementary figures and images for: The primary cilium functions as a mechanical and calcium signaling nexus
Source: Cilia. 2015 May 29;4:7. doi: 10.1186/s13630-015-0016-y (PMC4448211; doi:10.1186/s13630-015-0016-y)

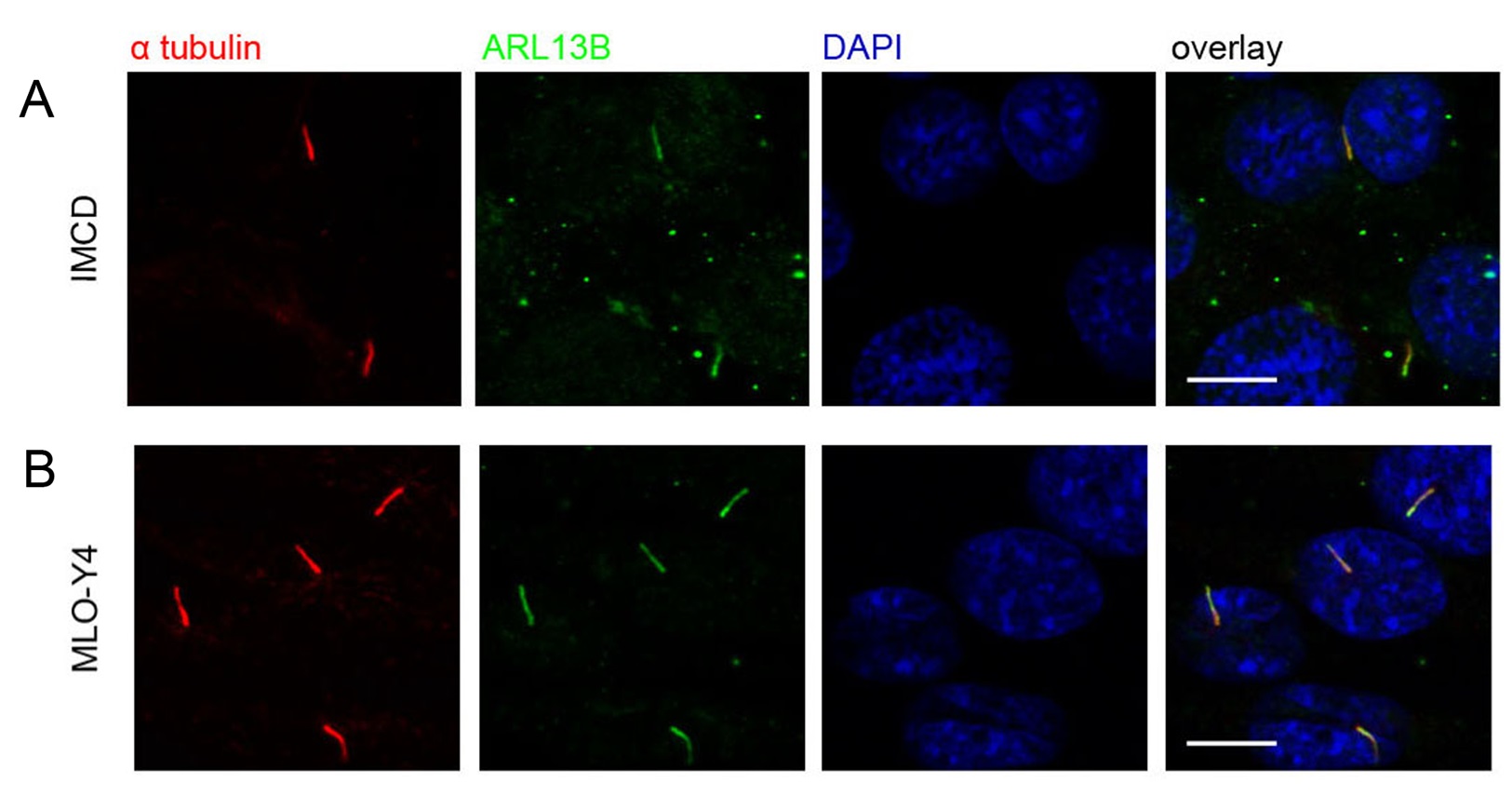

Supplement: Additional file 1: Figure S1. — ARL13B localizes to primary cilia in MLO-Y4 and IMCD cells. (A-B) IMCD and MLO-Y4 cells fixed and stained for acetylated alpha tubulin and ARL13B, demonstrating localization of ARL13B to the primary cilium. [file 13630_2015_16_MOESM1_ESM.jpeg]

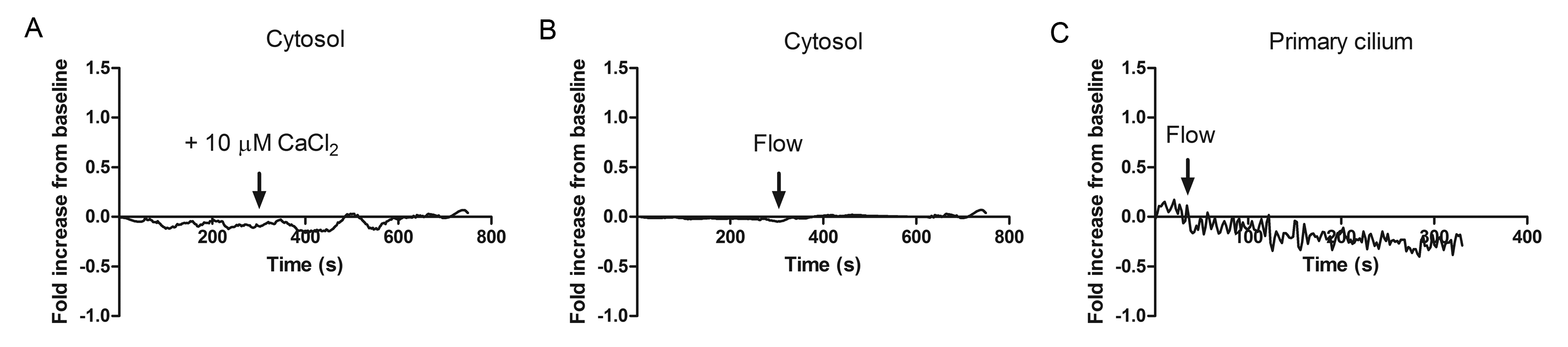

Supplement: Additional file 3: Figure S2. — Mutant CaB and ALC do not exhibit flow-induced Ca2+ peaks. Deletions of Trp3 and Phe17 in the M13 region inhibit FRET changes during Ca2+ increases. (A-B) In initial testing and prior to targeting the mutated biosensor to the primary cilium, MutCaB failed to detect flow-induced Ca2+ increase with the addition of 10 μM CaCl2 (n = 3) and steady flow (5 dyn/cm2) (n = 2). (C) MutALC failed to detect flow-induced Ca2+ increases occurring with oscillatory fluid flow (10 dyn/cm2) (n = 4). [file 13630_2015_16_MOESM3_ESM.tiff]

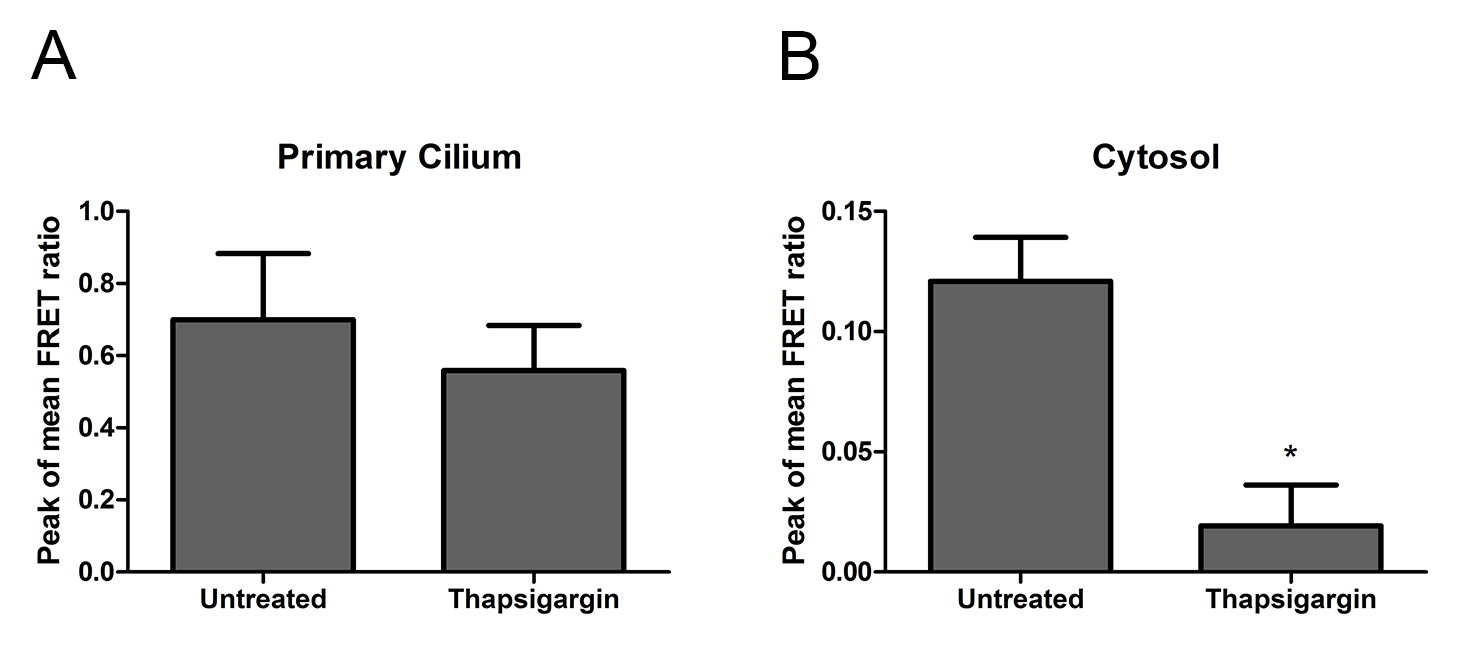

Supplement: Additional file 4: Figure S3. — MLO-Y4 cells express Piezo1 and not Piezo2. (A) Piezo1 and (B) Piezo2 mRNA levels in MLO-Y4 cells, IMCD cells, and adult murine (C57BL/6) brain and heart relative to GAPDH mRNA expression (n = 4–5). Piezo1 = 1.6 ± 0.2 and Piezo2 = 0.003 ± 0.001 in MLO-Y4 cells. Error bars show ±SEM. [file 13630_2015_16_MOESM4_ESM.tiff]

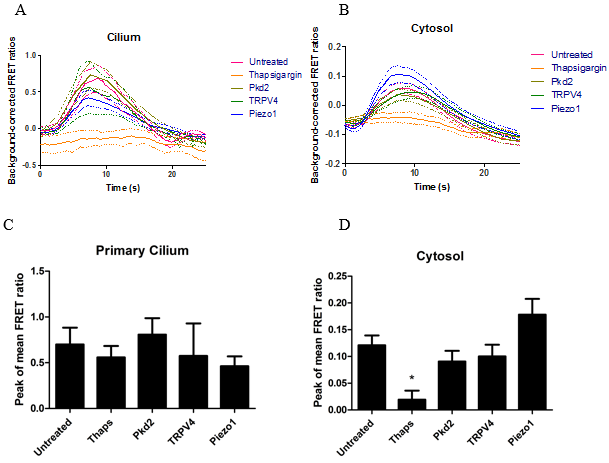

Supplement: Additional file 5: Figure S4. — FRET ratios of all viable MLO-Y4 cells exposed to fluid flow. (A) Mean FRET ratios over time, where solid lines indicate the mean background-corrected FRET ratios (no threshold applied) while the dashed lines indicate SEM. (B) Flow-induced Ca2+ peak amplitude averaged from all viable cells (no threshold applied). Error bars show ±SEM. [file 13630_2015_16_MOESM5_ESM.tiff]
